# Optimizing document retrieval using massive text embeddings and LLM prompt engineering

**DOI:** 10.1186/s13643-026-03155-4

**Published:** 2026-04-14

**Authors:** Goran Mitrov, Boris Stanoev, Vladimir Trajkovik, Biljana Risteska Stojkoska, Lasko Basnarkov, Petre Lameski, Martin Kampel, Eftim Zdravevski

**Affiliations:** 1 Faculty of Computer Science and Engineering, Ss. Cyril and Methodius University, Rugjer Boshkovik 16, Skopje, 1000 North Macedonia; 2 Magix.AI, Cyril and Methodius 3A, Skopje, 1000 North Macedonia; 3 Computer Vision Lab, TU Wien, Favoritenstr. 9/193-1, Vienna, 1040 Austria

**Keywords:** Massive text embeddings, LLMs, Prompt engineering, Document retrieval, Information retrieval, Vector indexes, Systematic review automation, Automated surveys

## Abstract

**Background:**

The rapid expansion of digital data poses a unique challenge for retrieving relevant and insightful information efficiently. In particular, the increasing volume of scientific publications has made literature reviews time-consuming. The emergence of large language models (LLMs) offers new opportunities to streamline this process.

**Methods:**

This paper explores the use of generative artificial intelligence (GenAI) for query reformulation and evaluates the performance of nine massive text embedding models, varying in size and fine-tuning strategies, in the context of document retrieval. We apply multiple prompt engineering techniques to evaluate the ability of LLMs to generate effective queries, comparing them with human-crafted queries. These are used to retrieve documents utilizing nine embedding models. The evaluation is across five datasets using metrics such as recall, average precision, and rank-based measures.

**Results:**

Results show that embedding models fine-tuned for semantic similarity consistently outperform general-purpose models, with UAE Large proving most robust across diverse domains. Furthermore, queries generated using zero-shot and few-shot prompting techniques often surpass the performance of human-formulated queries.

**Conclusion:**

These findings highlight the value of integrating LLMs and massive text embeddings to reduce manual effort in literature reviews. GenAI provides a reliable starting point for query formulation, with human input reserved for refinement when needed.

## Introduction

In an era of unprecedented data growth and an overwhelming flood of information, the ability to sift through vast amounts of raw documents and quickly extract the most relevant insights has become a critical need. Consequently, Information Retrieval (IR) systems play a crucial role in facilitating efficient access to desired resources. Recent advances in Natural Language Processing (NLP) have significantly enhanced the performance of IR systems by improving their ability to understand and interpret the complexities of natural language [1].

At the core of many IR applications is document retrieval, a process that provides a ranked list of relevant documents in response to a user’s query. This process is widely used across various fields, such as healthcare, law, and business, where quick access to pertinent information is crucial. In academia, whether researchers seek to quickly explore a specific niche or gain a broad overview of their field, document retrieval is a key component in efficiently conducting literature reviews [2]. Following the broader trend of data explosion in this digital era, the volume of scientific publications has been steadily increasing, with an annual growth rate of approximately 4% [3]. As a result, to navigate this vast sea of publications, the past decade has seen a growing number of review types, each incorporating different adaptations of the document retrieval process [4]. Most modern digital libraries offer various search functionalities, but they still face significant challenges. These include retrieving semantically relevant documents, as keyword-based search methods remain the dominant approach, handling ambiguous or vague user queries, and supporting the synthesis of relevant findings. As a result, much of the labor-intensive work is still left to researchers. The processes of database searching and paper selection during literature reviews are among the most time-consuming tasks, with experts highlighting these areas as the ones most in need of improved tool support [5].

As the volume of digital textual data has rapidly increased, the advancements in NLP techniques have kept pace. Over the past decade, we have seen the rise of text embeddings, which represent words as numerical vectors, followed by the development of transformer architecture, capable of capturing long-range dependencies in text. Most recently, we have witnessed the revolutionary emergence of large language models (LLMs) and their ability to comprehend and generate human language at an unprecedented scale. LLMs have made an immediate impact due to their power and versatility, demonstrating the ability to solve a wide range of tasks and showing immense potential for transforming fields such as medicine, education, finance, engineering, law, and more [6, 7]. LLMs have fundamentally transformed IR by enhancing each step of the document retrieval process, from improving query understanding and retrieval accuracy to enabling a more refined re-ranking of results, ultimately enriching the user experience with more context-sensitive and semantically relevant document matches [8]. One opportunity to improve IR is to assist users in constructing an ideal query. Zhai [9] claims that LLMs can bridge the vocabulary gap between queries and documents, clarify user intent, and assist in query transformation or reformulation.

We focus on literature reviews because they represent a challenging document retrieval task, involving high-recall search, ambiguous query formulation, and domain-specific semantic relevance. While this study is centered on this use case, the techniques we explore are applicable to a wide range of document retrieval scenarios. In the field of automating document retrieval for literature reviews, many existing tools utilize techniques from NLP and machine learning to assist researchers [10, 11]. However, LLMs offer the potential to further optimize these processes by improving accuracy and scalability while also holding promise for better integration into literature review workflows—motivating the exploration of their current capabilities in this area [12].

**Fig. 1:**
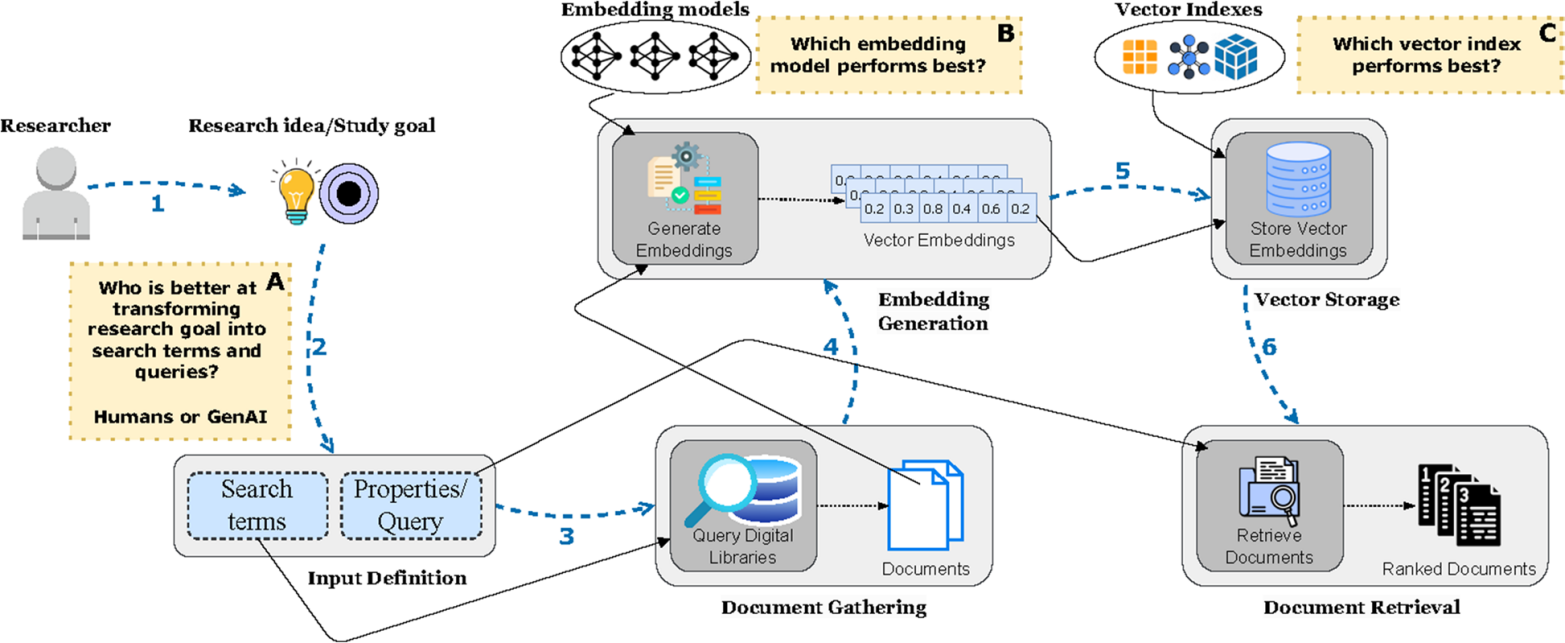
A high-level overview of the framework architecture

In previous work, we explored automating literature reviews by focusing on keyword-matching between paper abstracts and specific study properties [13]. However, this approach was limited by its reliance on exact lexical overlap, which often failed to capture the broader semantic context and nuanced terminology of diverse scientific domains. Building on this foundation, this study aims to integrate state-of-the-art technologies, such as massive text embeddings, LLMs, and vector databases, to enhance the capabilities and expand the range of functionalities. Figure 1 presents a high-level overview of our framework, visually summarizing the entire flow from the researcher’s initial idea to the retrieval of ranked scientific publications alongside the specific research goals labeled A, B, and C. The process begins with the researcher’s idea, goal, and aim, which are translated into an input consisting of search terms and properties (specific words and phrases acting as queries in the document retrieval process). The search terms are used to query digital libraries such as IEEE Xplore, Springer, MDPI, PubMed, and ScienceDirect, gathering an initial set of documents. We extract and clean the titles and abstracts from these documents, which are then embedded using massive text embeddings and stored in a vector database. Finally, the query is executed to retrieve and rank the most relevant documents.

In our study, we will primarily focus on three key research question, each targeting a critical part of the retrieval pipeline. The first aim (label A) is to investigate the differences between human experts and Generative AI in transforming a research goal into search terms and queries. With this, we want to examine whether, and in which scenarios, LLMs can assist in reformulating queries. We will compare the performance of human experts with three distinct prompt engineering techniques, measuring precision, recall, and the time spent on the retrieval process. Understanding the differences is crucial for designing assistive systems that enhance research productivity. Our second goal (label B) is to compare multiple massive text embeddings and determine which model performs best in the context of our use case. We investigate how variations in model size, training types, and fine-tuning strategies impact semantic similarity performance in the document retrieval process. Lastly, we will assess the impact of different storage options by comparing three vector indexes based on time and memory performance metrics and their effect on precision and recall (label C).

The remainder of this article is structured as follows: Subsection Related works reviews related work in information retrieval, large language modes, and their integration. Section Background introduces the foundational concepts essential to our study. Section Methodology outlines our research process, detailing the datasets, methodology, and experimental setup. Section Results presents the main results of our experiments, followed by a discussion in Section Discussion that highlights key findings and insights. Finally, Section Conclusion concluded our work.

### Related works

Information retrieval has been an essential field for decades, emerging nearly 70 years ago. Since its inception, it has steadily grown and evolved, beginning with foundational techniques like indexing, retrieval, TF-IDF, keyword-based search engines, and over time, integrating machine learning techniques [14]. Today, like many other fields, IR is experiencing exponential growth due to the enormous amount of data generated every second and the continuous breakthroughs in artificial intelligence (AI). In a recent study, [1] provide a comprehensive overview of modern IR systems, demonstrating that recent advancements in deep learning techniques, large labeled datasets, and increased computing power have significantly improved the systems’ performance and made them more capable of handling the complexity of natural language queries. In their latest research, [15] discuss generative information retrieval (GenIR) as a new direction in IR. They present GenIR as a novel retrieval paradigm that shifts from traditional document retrieval using indexes, and instead uses a generative model to encode documents into its internal parameters, offering deeper semantic understanding. However, the limitations of these models include challenges with scalability, memory capacity, and training complexity, and they are beyond the scope of our research.

The use of LLMs in IR can be explored from multiple angles. For instance, [16] provide an extensive review on the development and applications of dense retrieval models that leverage pre-trained LLMs to encode text into dense vectors, enabling more contextually rich retrieval. Similarly, [17] discuss integrating the generative capabilities of LLMs with traditional retrieval techniques to address challenges such as computational efficiency, factual accuracy, and domain-specific adaptability. They propose a paradigm that combines LLMs, IR models, and human input to enhance user modeling, dynamic indexing, and semantic matching. While these works establish a vital theoretical framework, our study moves beyond the theoretical paradigms to provide cross-domain validation of multiple text embedding models, testing the practical accuracy and scalability.

The nature of LLMs is to use statistical patterns and likelihoods to generate responses, and they are designed to serve as general-purpose models. As their outputs may sometimes lack specificity or relevance, prompt engineering emerged as a process to guide and refine LLM output, addressing these inherent limitations and maximizing their utility and accuracy [18]. Azad and Deepak [19] in their survey, provide an in-depth historical overview of query expansion (QE) methods and their impact on IR, categorizing the techniques into manual, automatic, and interactive approaches. As part of automatic query expansion approaches, [20] propose using LLMs to aid in query expansion, concluding that their application shows promising results and can improve retrieval performance metrics such as recall, mean reciprocal rank (MRR), and normalized discounted cumulative gain (NDCG). Acknowledging these studies, we adopt LLM-based query expansion using multiple prompting techniques to evaluate whether automated queries can mitigate the inherent limitations of human subjectivity in the document retrieval process.

Vector databases and LLMs work in synergy, with vector databases providing efficient methods for storing, retrieving, and managing the high-dimensional vectors intrinsic to LLM operations. When acting as a cost-effective semantic cache and a robust memory layer, vector databases can address several challenges LLMs face, such as hallucinations, high commercial application costs, and memory limitations [21]. To accelerate the IR process in high-dimensional embedding spaces and enable efficient lookup in large-scale environments, vector databases support indexes that implement approximate nearest neighbor (ANN) techniques to efficiently identify the closest points, improving speed and reducing computational costs. Aumüller et al. [22] present a benchmarking tool for evaluating these ANN indexes, assessing their performance and quality across standard datasets. In our study, the evaluations helped in the selection of our vector indexing platform, ensuring the chosen architecture maintained the necessary balance between computational efficiency and high retrieval accuracy in terms of requirements for systematic reviews.

In recent years, the automation and semi-automation of conducting literature reviews have gained momentum, with numerous researchers exploring the area using NLP, machine learning, and text-mining techniques [23–26]. In their study, [27] explored the process of conducting a literature review using AI, reporting a positive experience with approximately 77% of time saved. However, the highlight is also the need for specific remedies to address scenarios that could compromise the methodological quality of the review. Dennstädt et al. [28] utilized an LLM by constructing an instructional prompt that included the title, abstract, and relevance criteria to evaluate whether an article should be included in a literature review. They measured accuracy, recall, and precision based on the classification of articles, and their findings indicated promising results. However, such approaches often rely on binary classification for full automation, which carries the inherent risk of excluding relevant studies without human oversight. In contrast, we focus on identifying the most semantically relevant document through optimized retrieval and ranking, providing a decision-support tool that empowers the human researcher. Similarly, [29] developed an R package designed to automate the title and abstract screening process using GPT-4. In the validation process, the package demonstrated an overall accuracy of 84%, with specificities and sensitivities of 89% and 71%, respectively, when compared to human consensus decisions. In a complementary study, [30] evaluated the effectiveness of GPT-4 in identifying relevant titles and abstracts from real-world clinical review datasets, comparing its performance against ground truth labels provided by two independent human reviewers. They concluded that using LLMs as a support tool rather than a replacement can lead to more accurate and reliable conclusions in medical research. These studies underscore the potential of LLMs and AI-assisted tools in automating literature reviews. However, challenges such as optimizing accuracy, scalability, and domain adaptability remain, highlighting the need for continued research and innovation in this space.

## Background

In this section, we provide a brief technical background on the key concepts explored in our study. Specifically, we will cover text embeddings and their underlying architecture, vector indexes and databases, large language models (LLMs), and prompt engineering techniques.

### Embeddings

Embeddings are the transformation of unstructured data, such as text, into a structured format by mapping the semantic meaning of words, phrases, or entire documents into a continuous vector space of numerical values. Word embeddings, developed to capture semantic meaning and context, project words as vectors into a multi-dimensional space, where the distance and direction between vectors reflect the similarity and relationships among words [31, 32]. Text embeddings extend the concept of capturing contextual meaning to larger units of text, such as sentences or paragraphs. The emergence of transformer architecture marked a significant breakthrough, as its self-attention mechanism captures long-range dependencies and bidirectional context [33]. Massive text embeddings are large-scale, contextually rich representations generated from vast corpora of text using pre-trained models with millions of parameters, and they play a crucial role in our research.

### Vector databases and vector indexes

The increased use of embeddings in NLP, combined with the limitations of traditional databases in handling high-dimensional data, has led to the development of specialized data structures known as vector databases. These vector databases are optimized for storing high-dimensional vector data and enabling fast, efficient, and scalable searches [34]. Their storage capabilities and retrieval techniques make them well-suited for a wide range of applications, including recommendation systems, text generation and augmentation, and efficient document retrieval [35].

At the core of vector databases are vector indexes, which are structures designed to optimize similarity searches in high-dimensional spaces by enabling quick retrieval of vectors most similar to a query vector. These indexes support approximate nearest neighbor (ANN) search, a technique that significantly speeds up searches in large, high-dimensional datasets by finding neighbors close to the query with a certain level of approximation [36]. When comparing vector indexes, the three key parameters for evaluation are accuracy, time, and memory, and the challenge lies in finding the right balance between them. Our research will focus on three specific vector indexes: the flat index, the inverted file index (IVF), and the product quantization index (PQ).

**Fig. 2:**
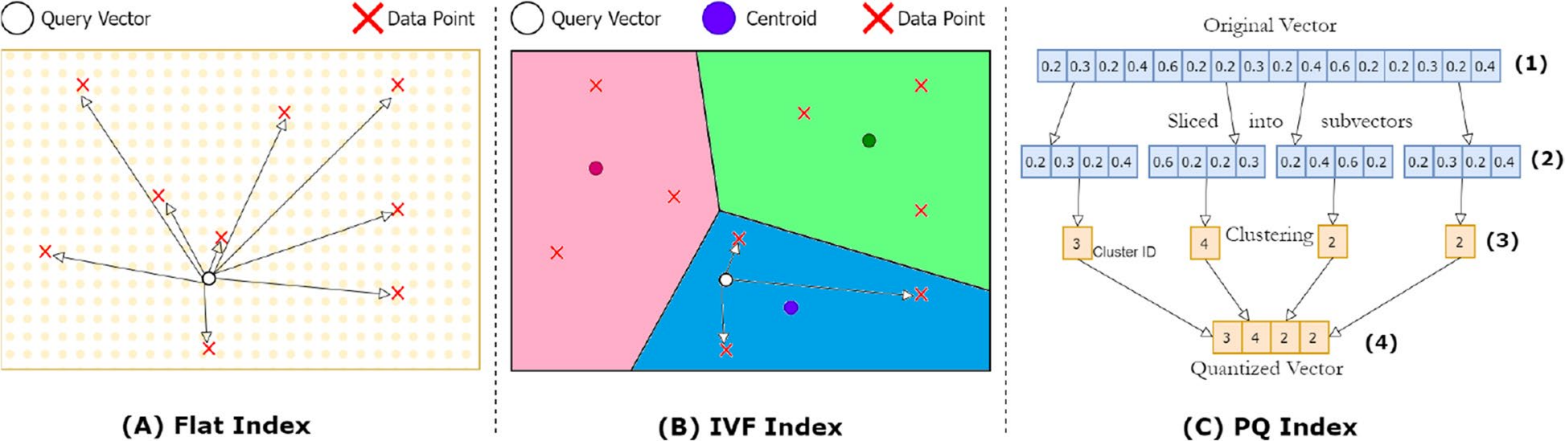
Overview of the vector index types

The flat index is the simplest vector index, performing a brute-force exhaustive search by calculating the distance between the query vector and all data points. It is highly accurate and requires no training, but it is computationally expensive and does not scale well. Figure 2A illustrates the search process using the flat index.

The IVF index speeds up the search process by reducing the search scope and using approximation. It partitions the vector space by clustering vectors into a predefined number of groups, finding centroids, and constructing a Voronoi cell diagram where each data point belongs to one cell [37]. This is illustrated in Fig. 2B. During the search, the query vector is mapped to a cluster, and the closest neighbors are searched within that cluster or neighboring clusters rather than across the entire dataset. This significantly reduces search time, although it requires initial training, and may affect accuracy depending on the clustering quality.

Product quantization (PQ) compresses high-dimensional data into a smaller space to reduce the memory footprint of indexes [38]. As shown in Fig. 2C, the PQ process begins by splitting the original vector into smaller sub-vectors. Each sub-vector is placed into its own subspace, along with sub-vectors from other original vectors. In each subspace, clustering is performed, and the sub-vectors are mapped to specific cluster centroids. The centroid values are then replaced with unique IDs, and the subspaces are merged to form the quantized vector. This method improves both speed and memory efficiency but sacrifices some accuracy due to lossy compression.

### Large language models

LLMs represent the most significant breakthrough in NLP, with the ability to understand, process, and generate human language. Predominantly built on transformer-based architecture, these models rely on massive text embeddings and learn complex semantic relationships from vast amounts of data through self-supervised and semi-supervised training processes [39]. The number of parameters in LLMs is measured in billions, and they are pre-trained on massive datasets containing general world knowledge. These models can also be fine-tuned on specific datasets and tasks, allowing them to adapt to particular applications and domains. Some of the most prominent and best-performing models include the GPT family [40], Gemini [41], LLaMA [42], Claude [43], Mistral [44], Gemma [45], and others [46]. LLMs are a subset of generative artificial intelligence (GenAI), computational techniques capable of producing original and meaningful content, such as text, images, audio, or code, that often seems indistinguishable from what humans might produce [47]. We will leverage the GenAI capabilities of LLMs in our research by applying various prompt engineering techniques for query expansion and query reformulation.

### Prompt engineering techniques

Communication with LLMs is done through a prompt, which is the input text provided to the model to obtain a specific response or prompt the model to perform a task. A prompt usually includes an instruction, along with optional components such as context, additional background information to guide the response, input data, and output indicators or constraints. Prompt engineering is an iterative process of refining prompts to guide LLMs toward more accurate and relevant responses. A prompt engineering technique is a strategy for structuring the input so that the model generates more accurate and relevant responses [48]. The field of prompt engineering is thriving, with numerous diverse methods and techniques, each with its own methodology and application [49]. Our research will focus on three prompt engineering techniques: zero-shot, few-shot, and prompt chaining.

Zero-shot prompting eliminates the need for training data and relies on prompts that ask the model to perform a task without providing examples to guide the response. This requires the model to generate a response based solely on its pre-existing knowledge [50]. Few-shot prompting is a technique that enables in-context learning by providing a few input-output examples to guide the model in understanding the task pattern, unlike zero-shot prompting [51]. Prompt chaining is useful for accomplishing complex tasks that require detailed prompts. With this technique, the task is broken down into sub-tasks, each performed in a separate prompt, with the output of one prompt serving as the input for the next until the desired outcome is achieved.

## Methodology

In this section, we present our research methodology, beginning with an overview of the datasets used in our study. We then discuss the selection of massive text embedding models, the design and implementation of prompts, and the choice of vector indexes. Following this, we describe the full experimental setup and conclude with the evaluation metrics we employ to assess the performance.

### Datasets

The datasets used in this study were obtained through close collaboration with domain experts conducting real research studies, during which they utilized our NLP tool. Beginning with their initial ideas and research goals defined in a textual form, and leveraging their domain-specific knowledge, we work together to reformulate these goals into inputs suitable for the tool. This includes deriving keyword-based search strings to query the digital libraries and defining semantic “properties” used to construct the query vector for ranking the documents. Using the search strings, the tool gathers a set of candidate documents containing information such as DOI, title, abstract, and other relevant details. This pool of documents, referred to as *papers provided* in Table 1, is then presented to the researchers for evaluation. Through a thorough manual review process, the experts select the documents they consider relevant for inclusion in their research. These selected documents, listed as *papers selected* in Table 1, serve as the labeled ground-truth for evaluating retrieval performance in our study. In Table 1, we provide an overview of the datasets, including the number of papers initially available to the researchers and the final number of articles selected as relevant.

**Table 1:** Datasets overview

Dataset	Papers provided	Papers selected	Reference
Driver healthcare monitoring	13,518	30	Awaiting publication
Venture capital	17,133	150	Awaiting publication
Relational learning	18,711	23	[52]
Ambient assisted living	26,331	108	[53]
10-m walks	6,708	22	[54]

The following is a detailed overview of the datasets, including their origin and focus areas:

- Driving healthcare monitoring with IoT and wearable devices: a systematic review—this dataset is derived from a systematic review exploring the use of IoT and wearable devices in monitoring drivers’ health.
- Venture capital: a bibliometric analysis—this dataset is obtained from a bibliometric and structural review and highlights three primary topics: environmental, social, and governance (ESG) factors, innovation, and exit strategies within the venture capital field.
- Automating feature extraction from entity-relation models: experimental evaluation of machine learning methods for relational learning—this dataset is curated from a study that included a comprehensive review of the literature on relational learning and further explores machine learning methods for feature extraction from entity-relation models.
- Ambient assisted living (AAL): scoping review of artificial intelligence (AI) models, domains, technology, and concerns—this dataset originates from a comprehensive scoping review that identifies, analyzes, and extracts literature on AI models in AAL.
- Mobile and wearable technologies for the analysis of 10-m walk test: a concise, systematic review—this dataset is derived from a systematic review focused on using mobile and wearable devices to measure physical parameters during the 10-m walk test, analyzing test performance.

**Fig. 3:**
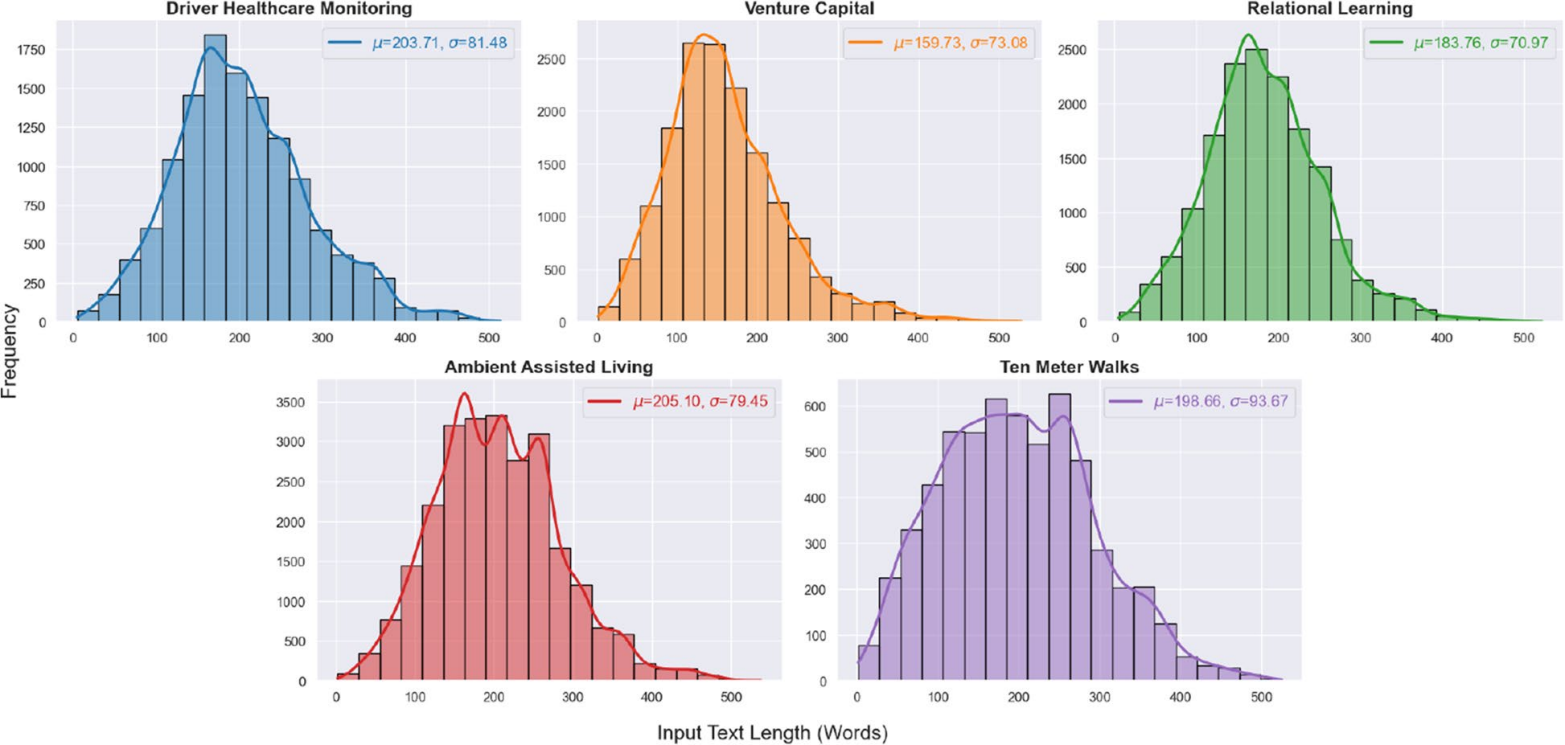
Histogram and distribution for the input document text length per dataset

At the start of our investigation, we conducted an exploratory analysis focusing on the length of the documents in each dataset. After cleaning the title and abstract of each article, we tokenized the text, treating each word as a separate token, and created a histogram to visualize the token count distribution, as illustrated in Fig. 3. The analysis reveals that all datasets exhibit an approximately normal distribution, with similar means and standard deviations, showing no statistically significant differences. This allows us to exclude document length as a factor when comparing metrics during embedding creation, which is crucial for scalability and comparing vector indexes in terms of time performance. The remaining differentiating factor between the datasets will be the number of documents.

### Massive text embeddings

This subsection provides an overview of the massive text embeddings used in our research. For the selection of the models, we consulted the massive text embedding benchmark (MTEB) [55] which evaluates models across diverse embedding tasks. Our goal was to include models that vary in size and functionality. We selected nine models based on their overall benchmark scores, as well as their performance in specific tasks such as retrieval and semantic text similarity. In Table 2, we present the key characteristics of each model, including the number of parameters (in millions), memory usage (in GB), the number of dimensions, and the average model loading time (in seconds).

**Table 2:** Overview of the massive text embedding models

Embedding model	Number of parameters (millions)	Memory usage (GB)	Number of dimensions	Average load time (seconds)
MiniLM	23	0.085	384	2.36
BGE Large v1.5	335	1.249	1024	2.52
BGE M3	560	2.11	1024	4.48
E5 Large v2	335	1.25	1024	3.32
UAE Large	335	1.25	1024	3.05
Snowflake Arctic L	334	1.24	1024	3.30
QWEN2	1776	9.25	1536	6.65
Stella v5	1543	9.25	1024	9.40
E5 Mistral	7111	26.49	4096	13.79

Furthermore, Table 3 complements the previous table by providing a qualitative analysis of the training methodologies, the types of data the models were trained on, and the specific strengths or tasks for which they are best suited. Through this, we aim to examine the qualitative aspects of the models and offer deeper insight into the underlying factors that may explain the performance variations.

**Table 3:** Qualitative comparison of the massive text embedding models

Embedding model	Training type	Training data domain	Strengths/use cases
MiniLM [56, 57]	$\bullet$ Teacher-student distillation	$\bullet$ Open-domain text	$\bullet$ Low-resource environments
	$\bullet$ Contrastive learning	$\bullet$ Sentence pairs	$\bullet$ Sentence similarity
			$\bullet$ Quick inference
BGE Large v1.5 [58]	$\bullet$ Contrastive learning	$\bullet$ General web corpora	$\bullet$ Semantic search
	$\bullet$ Task-specific fine tuning	$\bullet$ Curated QA datasets	$\bullet$ Document ranking
			$\bullet$ Versatility
BGE M3 [59]	$\bullet$ Multi-stage training	$\bullet$ Multi-lingual	$\bullet$ Cross-lingual search
	$\bullet$ Self-knowledge distillation	$\bullet$ Multi-format retrieval data	$\bullet$ Hybrid retrieval
E5 Large v2 [60]	$\bullet$ Contrastive learning	$\bullet$ CCPairs (query-passage)	$\bullet$ Question answering
		$\bullet$ QA pairs	$\bullet$ Re-ranking
UAE Large [61]	$\bullet$ Contrastive learning	$\bullet$ General text	$\bullet$ Fine-grained semantic similarity
	$\bullet$ Angle optimization	$\bullet$ Semantic similarity tuning	$\bullet$ Robust generalization
Snowflake Arctic L [62]	$\bullet$ Contrastive learning	$\bullet$ Stratified IR corpora	$\bullet$ Scalable IR tasks
	$\bullet$ Retrieval-specific fine-tuning	$\bullet$ Enterprise datasets	
QWEN2 1.5B Instruct [63]	$\bullet$ Contrastive learning	$\bullet$ Code	$\bullet$ Instruction-following tasks
	$\bullet$ Instruction tuning	$\bullet$ Forums	$\bullet$ Classification
		$\bullet$ Social media	
Stella 1.5B v5 [64]	$\bullet$ Matryoshka Representation Learning	$\bullet$ Open-domain multi-task sets	$\bullet$ Multi-task
E5 Mistral 7B Instruct [44, 65]	$\bullet$ Contrastive learning	$\bullet$ Multilingual text corpora	$\bullet$ Multilingual IR
	$\bullet$ Multilingual fine tuning		$\bullet$ Document re-ranking

### Prompt engineering techniques

In this subsection, we provide the implementation details for the prompt engineering techniques used in our study. As shown in Fig. 1, the second step of our framework involves transforming the researcher’s study goal into search terms and queries for the document retrieval process in a format suitable for our NLP tool. Initially, this query reformulation was conducted manually, relying on researcher expertise, which made the process time-consuming. To address this, we introduce GenAI to automate the query reformulation phase, exploring three prompt engineering techniques: zero-shot, few-shot, and prompt chaining. These techniques are widely used, effective, and relatively straightforward to implement.

In designing our prompts, we followed the best practices recognized by the community, including the clear separation of instruction, context, and input data, specifying the desired length and format of the output, and avoiding ambiguity by providing clear handling for edge cases.

#### Zero-shot prompting

Zero-shot prompting refers to the technique in which a prompt is provided to the model without any examples or demonstrations. The model is instructed to perform the task based solely on the given instruction, without additional examples to guide its response. In our implementation, the prompt consists of an instruction, context, desired output format, and the input data. Below is the instruction used in our zero-shot prompting approach.
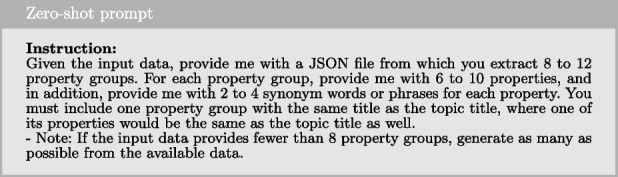


To provide the model with further guidance on how to handle the instruction, we include additional context, which is demonstrated below.
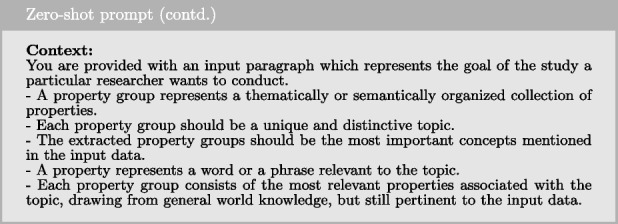


Next, we provide explicit guidance on the model’s expected outcome, as outlined below.
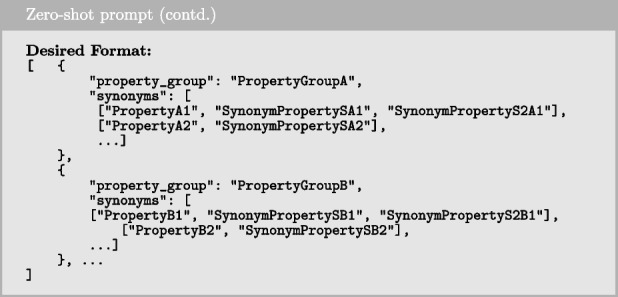


Together with the study goal as input data, these elements form the complete prompt sent to the LLM to generate a response.

#### Few-shot prompting

Few-shot prompting is a technique that builds upon zero-shot prompting by enabling in-context learning, where we provide demonstrations to guide the model toward better performance. These examples serve as conditioning for subsequent instances where we want the model to generate a response. In our use case, we utilized the same prompt structure as in zero-shot prompting but added four examples (one from each of the remaining datasets), making it 4-shot prompting. In each example, we used the study goal as the input and the ground truth query constructed by the researcher as the output. The examples were formatted as shown below.
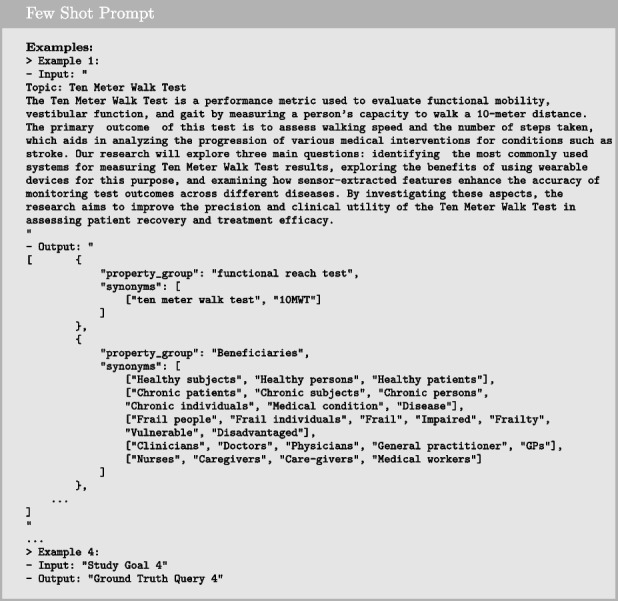


#### Prompt chaining

Prompt chaining is a technique to accomplish complex tasks that an LLM might struggle to handle effectively when given a single, detailed prompt. This approach divides the task into multiple subtasks, and the LLM is first prompted with one subtask. Its response is then used as input for the next prompt, creating a chain of prompts. In our use case, we address the complex task of query reformulation by breaking it into three subtasks: extraction, expansion, and formatting. We handle these transformations in separate prompts to achieve the final desired output.

We begin with the first prompt by providing the initial instruction to extract information from the input, as demonstrated below:
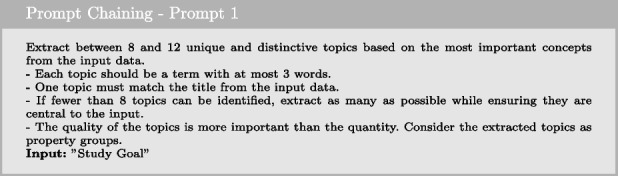


Next, we use the LLM’s output from the first prompt to provide additional instruction for expanding on the extracted information, as shown below:
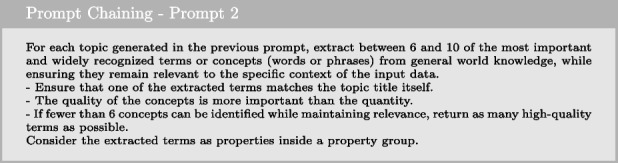


We conclude the chain with the final prompt, where we finalize the content of the desired outcome and provide specific formatting for the response.
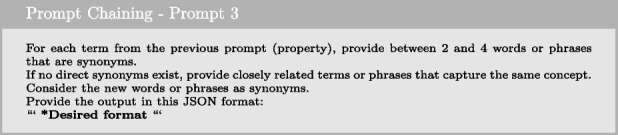


### Vector indexes

In this subsection, we provide the implementation details for the vector indexes used in our research. These indexes store the vector embeddings generated by the massive text embedding models and retrieve ranked documents when a query is made. To focus on comparing the performance of different types of indexes and avoid potential overhead introduced by vector databases, we chose to work with FAISS [66], a lightweight library for efficient similarity search on dense vectors. For this research, we selected the flat index as a baseline, along with two additional indexes: the IVF index to compare the trade-off between time consumption and performance and the PQ index to examine the trade-off between memory usage and performance.

#### Flat index

For the flat index implementation, we use the FAISS *IndexFlatL2* with the number of vector dimensions as the only parameter. This implementation encodes the vectors into fixed-size codes and stores them in an array without compression or additional overhead. During the search phase, an exhaustive brute-force search is performed, where all indexed vectors are decoded sequentially and compared to the query vector using the L2 (Euclidian distance) metric.

#### Inverted file index (IVF)

For the IVF index, we use the *IndexIVFFlat* implementation, which requires a quantizer index, the number of vector dimensions, and the number of clusters. The quantizer groups data into clusters using k-means clustering and assigns each data point to a cluster during indexing. In the search process, it identifies the nearest clusters and searches within their vectors. We use the flat index as the quantizer, and for the number of clusters, we follow the library’s recommendation of using the square root of the total number of documents in the index.

During the search, we also adjust the *nprobe* parameter, which determines how many of the closest clusters are searched. We iteratively experimented to develop a dynamic formula for the parameter to ensure that at least N closest neighbors are found while searching the fewest clusters possible.

#### Product quantization index (PQ)

For the PQ index, we use the *IndexPQ* implementation, which requires the number of vector dimensions, the number of sub-vector splits, and the number of bits necessary to represent each sub-vector in its compressed form. We set the number of sub-vectors to *d*/8, where *d* is the vector dimension. This ensures a balanced sub-vector size and efficient quantization. For the *nbits*, we choose a value of 8, corresponding to 256 centroids per sub-vector space, providing sufficient granularity for approximation and effective memory optimization. Consequently, during a search, we do not directly compare the original high-dimensional vectors but instead operate in a compressed domain.

### Experiments

In this subsection, we describe the experimental setup, intermediate findings, and key observations made during the process without focusing on the final results. Our experiments began with clean datasets that included a document identifier, title, abstract, and label indicating whether the document was considered relevant in the corresponding research process. The experiments were divided into two phases: one for generating embeddings and the other for querying and retrieval.

All experiments, except those involving the Mistral model, were conducted on a server equipped with an NVIDIA TITAN V GPU, featuring 640 tensor cores, 5120 CUDA cores, and 12 GB of high-bandwidth memory (HBM). Due to the specific hardware requirements for the Mistral model, those experiments were performed on a cloud-based virtual machine with an NVIDIA A100 GPU, offering 640 tensor cores, 6912 CUDA cores, and 40 GB of HBM.

**Fig. 4:**
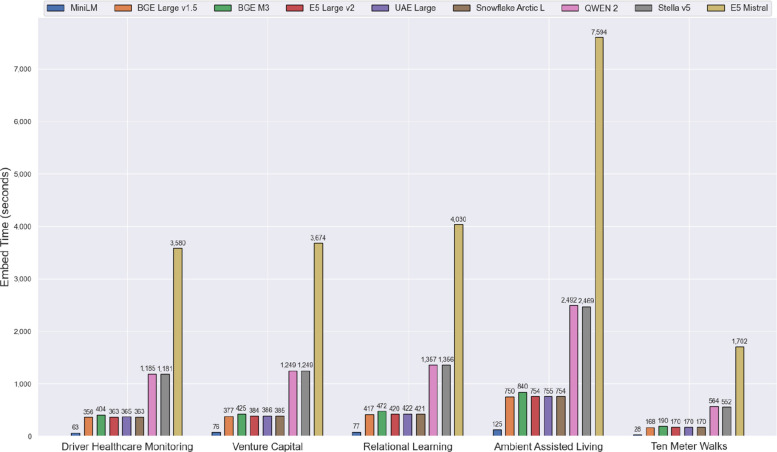
Clustered bar chart for times necessary for embedding generation per model and dataset

In the first phase, we iterated through each dataset and embedding model, generating vector embeddings for the documents and storing them as *.npy* files, the standard binary format in NumPy for persisting arrays on disk. As part of the embedding generation phase, we measured the time required to compute embeddings for each dataset using different text embedding models. The results, shown in Fig. 4, provide a comparative view of each model’s embedding times (in seconds) across the datasets.

MiniLM consistently exhibits the shortest times, while E5 Mistral requires the longest time due to differences in the number of parameters and the output vector embedding size. Using the time required for generating embeddings with MiniLM as a baseline, models such as BGE Large, BGE M3, E5 Large, UAE Large, and Snowflake Arctic L took approximately 5 to 6 times longer. QWEN2 and Stella required around 18 times more time, while Mistral took about 50 to 60 times longer for the same task. Our analysis also shows that embedding times increase linearly as a function of the number of documents. This suggests that the computational load scales predictably with dataset size, reinforcing the importance of selecting an appropriate model based on the size of the dataset and time constraints.

In the second phase, the process involved two steps. First, we loaded the pre-generated embeddings for each dataset and embedding model and created the three types of vector indexes. Additionally, we trained the indexes that required a training phase. During this step, we measured three main metrics: training time, build time (the time required to populate the indexes), and memory consumption. The average aggregated results of these metrics per dataset and index type are presented in Table 4.

**Table 4:** Time and memory requirements for building vector indexes

Dataset	Index type	Training time (s)	Build time (s)	Memory consumption (MB)
Driver healthcare monitor	Flat	0	0.031	65.42
	IVF	0.472	0.059	66.1
	PQ	10.553	0.399	3.36
Venture capital	Flat	0	0.027	82.48
	IVF	0.718	0.082	83.25
	PQ	13.332	0.499	3.89
Relational learning	Flat	0	0.036	82.17
	IVF	0.67	0.078	82.94
	PQ	13.28	0.498	3.88
Ambient assisted living	Flat	0	0.053	130.04
	IVF	1.3	0.148	131.05
	PQ	21.16	0.782	5.38
10-m ealks	Flat	0	0.014	30.43
	IVF	0.189	0.022	30.87
	PQ	5.234	0.199	2.27

The results reveal important insights into the trade-offs among the index types. All values for the build time are below 1 s, making their impact negligible. When using the flat index as a baseline, we observe that IVF introduces a small training overhead, ranging between 0 and 1 s, and consumes slightly more memory. While this might make IVF seem less efficient compared to the flat index in terms of these metrics, its primary advantage lies in reducing query times, which is not reflected in this table. On the other hand, the PQ index offers a significant reduction in memory consumption, approximately 20 times less than the flat index. However, this memory efficiency comes at the cost of increased training times, which range from 10 to 20 s in most cases.

**Fig. 5:**
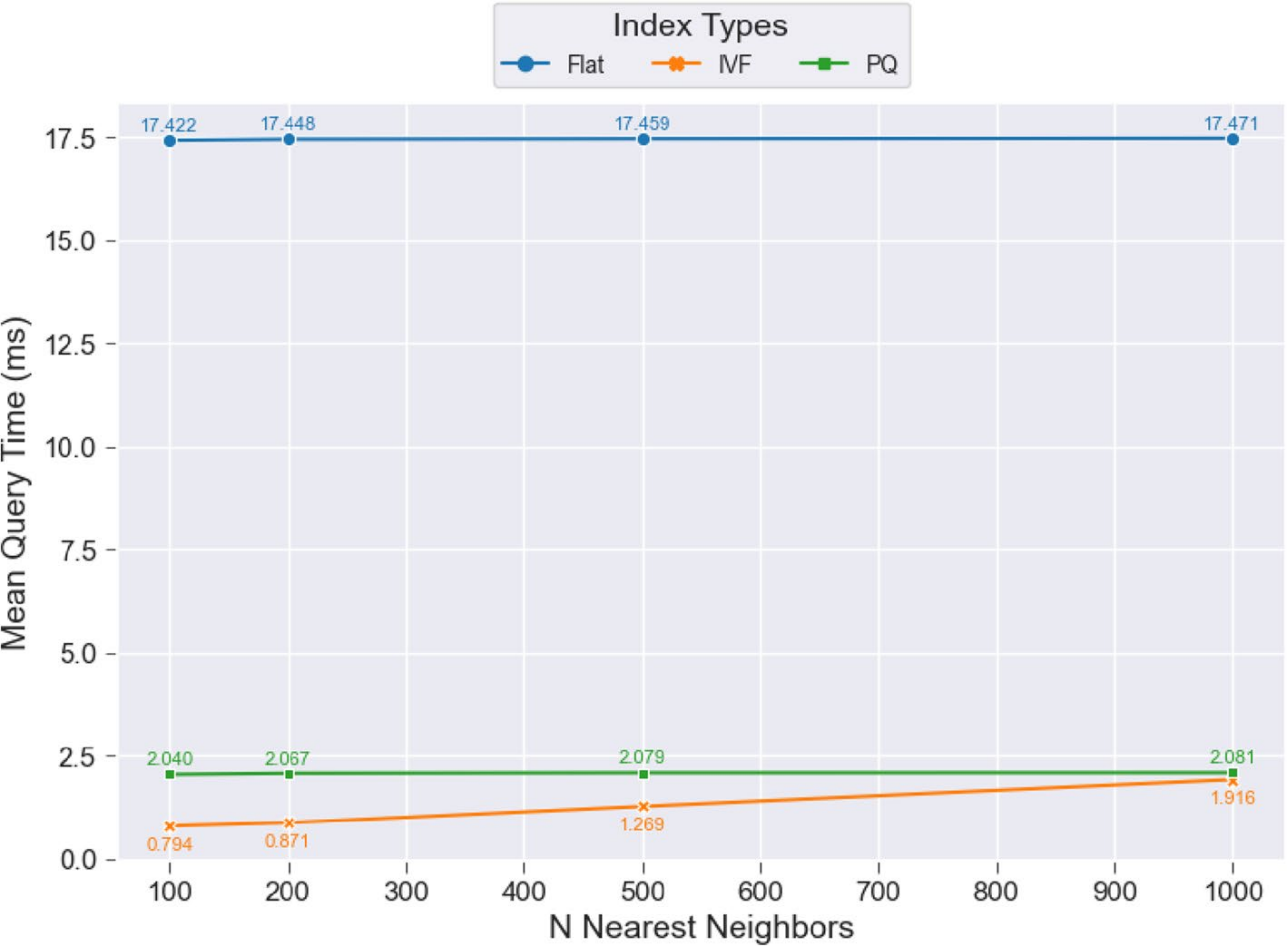
Mean query response time per index type

Finally, after setting up the indexes, we concluded our experiments by sending queries to the indexes and retrieving a ranked list of the closest documents. We experimented with different numbers of closest documents to retrieve (100, 200, 500, and 1000) to assess the performance of each index type. We also measured the time required for each index type to compare and return the results during this step. To evaluate the performance, we computed the mean query time for each index type across the datasets and embedding models, which is presented in Fig. 5.

From the figure, we can observe that both the Flat and PQ indexes have constant, straight lines because they perform an exhaustive search, regardless of the number of closest documents returned. This results in a fixed computational load for both indexes. However, the PQ index returns results approximately 8.5 times faster than the Flat index due to its operation in the compressed vector domain. On the other hand, the IVF index shows faster performance for the smaller number of closest documents, returning results in under 1 ms per query for 100 closest documents. As the number of closest documents increases, the query time gradually rises, matching the PQ index’s performance for 1000 documents.

### Evaluation metrics

In this subsection, we provide definitions for the evaluation metrics used to assess the performance of our document retrieval system, focusing on comparing the different embedding models and query formulations. Specifically, we use the order-unaware metric Recall@K, as well as order-aware metrics such as Average Precision@K (AP@K), Average Rank (AR), Median Rank (MR), and Reciprocal Rank (RR).

#### Recall@K

Recall@K (R@K) is one of the most widely used and interpretable evaluation metrics in IR systems. It measures the proportion of relevant documents retrieved out of all relevant documents in the entire dataset. In our use case, we assess how many of the relevant documents selected by a researcher were retrieved by the system for different values of K (at 100, 500, and 1000). If a researcher selected *N* relevant articles, and only *M* of them are among the top *K* results, the recall is calculated as

1 $$\begin{aligned} \text {Recall@K} = \frac{M}{N} \end{aligned}$$

#### Average Precision@K

Average Precision@K (AP@K) is an order-aware metric used to evaluate the relevance of a ranked list of retrieved documents. It measures the precision of the results up to a specific position, K, accounting for both the quality and ranking of relevant items within the result set. To calculate AP, the first step is to compute Precision@K, which measures the proportion of relevant documents out of the top K retrieved documents. If a researcher selected *N* relevant articles, and *M* of them appear among the top *K* results, the precision is calculated as:

2 $$\begin{aligned} \text {Precision@K} = \frac{M}{K} \end{aligned}$$

AP@K extends this by calculating the average of the precision values at each point where a relevant document appears in the ranked list of K documents, providing a more fine-grained assessment of the system’s ability to rank relevant documents higher. It is calculated as in Eq. 3, where $rel_k$ is a relevance parameter which is equal to 1 when $k^{th}$ item is relevant or 0 when it is not.

3 $$\begin{aligned} \text {Average Precision@K} = \frac{\sum _{k=1}^{K} \text {Precision@K} * rel_k}{M} \end{aligned}$$

#### Average rank

Average rank (AR) is a simple yet insightful metric that considers the entire ranked list of documents. It calculates the mean rank position of each relevant document, with a lower average rank indicating better retrieval effectiveness. If the researcher has selected *N* relevant documents, the value for this metric is calculated as in Eq. 4, where $D_i$ is the $i^{th}$ relevant document from the dataset.

4 $$\begin{aligned} \text {Average Rank} = \frac{\sum _{i=1}^{i=N} \text {Rank}(D_i)}{N} \end{aligned}$$

#### Median rank

Median rank (MR) is similar to AR, but it measures the central tendency of the ranks of relevant documents. It identifies the middle rank, providing a more robust measure of where the majority of relevant documents are located in the list. If the researcher has selected *N* relevant documents, the value for this metric is calculated as follows:

5 $$\begin{aligned} \text {Median Rank} = \left\{ \begin{array}{ll} \left( \frac{N + 1}{2}\right) ^{th} \text { term} & \text {if } N \text { is odd}, \\ \frac{\left( \frac{N}{2}\right) ^{th} \text { term } + \left( \frac{N + 1}{2}\right) ^{th} \text { term}}{2} & \text {if } N \text { is even}. \end{array}\right. \end{aligned}$$

We take into consideration both metrics, with AR offering a holistic view of how relevant documents are distributed across the entire ranked list, whereas MR highlights where most of the relevant documents tend to cluster.

#### Reciprocal rank

Reciprocal rank (RR) is an order-aware metric that measures how quickly the first relevant document appears in a ranked list of documents. It is calculated as the reciprocal of the rank at which the first relevant document is found. Mathematically, if the first relevant document appears at position *k*, the reciprocal rank is calculated as follows:

6 $$\begin{aligned} \text {Reciprocal Rank} = \frac{1}{k} \end{aligned}$$

In this study, we refrain from using mean reciprocal rank (MRR) since our research involves a single query per prompt engineering technique.

## Results

In this section, we present the results of our experiments, focusing on the performance of nine text embedding models across five datasets, using four different prompt engineering techniques. We provide a separate table for each prompt engineering technique showcasing the performance on the evaluation metrics defined in Subsection Evaluation metrics.

We begin by presenting the results for the embedding models utilizing the ground truth queries formulated by human experts, as outlined in Table 5. For the Driver Healthcare Monitor dataset, BGE Large and BGE M3 lead the performance across different metrics, each excelling in specific areas. In the Venture Capital and Relational Learning datasets, UAE Large emerges as the top performer, with BGE Large and MiniLM also showing strong results in some metrics. This trend of varying model performance continues with the Ambient Assisted Living dataset, where BGE Large delivers the highest scores. Finally, Snowflake Arctic L and UAE Large demonstrate superior performance for the 10-m walks dataset.

**Table 5:** Ground truth results

Dataset	Model	R@100	R@500	R@1000	AP@100	AP@500	AP@1000	AR	MR	RR
Driver healthcare monitor	MiniLM	0.033	0.2	0.267	0.032	0.014	0.013	2952	2584	0.032
	BGE Large	0.033	0.233	**0.5**	**0.333**	0.061	0.038	**1648**	**1071**	0.333
	BGE M3	**0.067**	**0.267**	0.4	0.306	**0.096**	**0.069**	2837	1821	**0.5**
	E5 Large	0	0.1	0.167	0	0.007	0.007	3983	4103	0.006
	UAE Large	0.033	0.2	0.467	0.167	0.043	0.027	1702	1162	0.167
	Snowflake Arctic L	0	0.067	0.233	0	0.005	0.007	4418	3929	0.004
	QWEN2	**0.067**	0.133	0.2	0	0	0.002	3327	2433	0.001
	Stella	0.033	0.167	0.333	0.012	0.012	0.012	3622	3045	0.012
	Mistral	**0.067**	0.167	0.267	0.019	0.016	0.014	2536	1836	0.017
Venture capital	MiniLM	0.093	**0.36**	0.547	0.206	**0.156**	**0.135**	1452	848	**1**
	BGE Large	0.093	0.273	0.447	0.128	0.105	0.094	1713	1087	0.2
	BGE M3	0.067	0.253	0.38	**0.326**	0.147	0.121	2389	1601	**1**
	E5 Large	0.053	0.2	0.307	0.295	0.126	0.1	2400	1848	**1**
	UAE Large	**0.1**	0.32	**0.567**	0.216	0.143	0.122	**1429**	**797**	0.5
	Snowflake Arctic L	0.06	0.22	0.38	0.21	0.115	0.093	2067	1392	**1**
	QWEN2	0.007	0.047	0.073	0.011	0.012	0.013	5542	5737	0.011
	Stella	0.093	0.2	0.293	0.24	0.154	0.121	3450	2356	0.5
	Mistral	0.06	0.3	0.507	0.133	0.104	0.097	1921	967	0.062
Relational learning	MiniLM	0.261	0.913	0.957	0.139	0.091	0.089	239	136	0.333
	BGE Large	**0.739**	**1**	**1**	0.276	0.235	0.235	81	**41**	0.143
	BGE M3	0	0.217	0.391	0	0.007	0.008	2173	1546	0.004
	E5 Large	0	0.348	0.739	0	0.012	0.015	1416	722	0.007
	UAE Large	**0.739**	**1**	**1**	0.272	**0.237**	**0.237**	**66**	**41**	0.167
	Snowflake Arctic L	0.435	0.826	0.913	0.124	0.101	0.094	321	140	0.077
	QWEN2	0	0.13	0.13	0	0.008	0.008	3963	3602	0.007
	Stella	0.13	0.217	0.435	**0.37**	0.226	0.118	2482	1223	**1**
	Mistral	0.217	0.478	0.609	0.117	0.077	0.064	1014	557	0.167
Ambient assisted living	MiniLM	0.046	0.185	0.269	0.179	0.082	0.067	3935	2534	0.5
	BGE Large	**0.102**	**0.296**	**0.407**	0.121	0.094	0.082	**2782**	**1383**	0.25
	BGE M3	0.046	0.13	0.204	0.195	0.096	0.07	3614	2637	0.333
	E5 Large	0.074	0.194	0.352	0.1	0.069	0.057	3091	1401	0.333
	UAE Large	0.065	0.259	0.333	0.105	0.079	0.071	3077	1740	0.25
	Snowflake Arctic L	0.028	0.093	0.176	0.112	0.052	0.037	4972	2961	0.111
	QWEN2	0.009	0.009	0.019	0.014	0.014	0.008	12576	13391	0.014
	Stella	0.019	0.056	0.111	**0.511**	**0.18**	**0.097**	7955	4840	**1**
	Mistral	0.046	0.102	0.213	0.332	0.167	0.094	3914	2777	**1**
10-m walks	MiniLM	0.091	0.545	0.773	0.022	0.022	0.022	679	433	0.02
	BGE Large	0.182	0.545	0.773	0.043	0.043	0.036	583	**357**	0.032
	BGE M3	0.091	0.364	0.636	0.036	0.029	0.023	1008	703	0.037
	E5 Large	0.045	0.273	0.591	0.024	0.012	0.013	980	778	0.024
	UAE Large	0.136	**0.636**	**0.818**	0.031	0.037	0.034	**581**	372	0.027
	Snowflake Arctic L	**0.318**	**0.636**	0.727	**0.061**	**0.051**	**0.048**	812	337	**0.056**
	QWEN2	0.045	0.045	0.091	0.019	0.019	0.008	1850	1328	0.019
	Stella	0	0	0.045	0	0	0.002	3003	3539	0.002
	Mistral	0.091	0.455	**0.818**	0.03	0.03	0.024	610	690	0.036

*R@K* Recall@K, *AP@K* Average Precision@K, *AR* average rank, *MR* median rank, *RR* reciprocal rank

Bold numbers = best choices within the table

In Table 6, we present the performance of the embedding models using queries generated by the zero-shot prompting technique. Our analysis shows that, for the Driver Healthcare Monitor dataset, BGE Large and BGE M3 stand out. BGE Large excels in recall, average rank, and median rank, while BGE M3 achieves higher performance in average precision.

In the Venture Capital dataset, we see a shift in the top performers, with Mistral achieving the best results for recall and rank metrics, and MiniLM leading in average precision. For the Relational Learning dataset, MiniLM dominates across most metrics, with other models also achieving near-perfect recall, approaching 100%.

In the final two datasets, Ambient Assisted Living and 10-m walks, Mistral clearly outperforms the other models, although BGE M3 stands out for its superior average precision in the 10-m walks dataset.

**Table 6:** Zero shot results

Dataset	Model	R@100	R@500	R@1000	AP@100	AP@500	AP@1000	AR	MR	RR
Driver healthcare monitor	MiniLM	0.067	0.1	0.267	0.035	0.026	0.015	1949	1518	0.038
	BGE Large	0.1	**0.467**	**0.767**	0.107	0.045	0.037	**906**	**617**	0.25
	BGE M3	0.067	0.233	0.367	**0.306**	**0.097**	**0.067**	1850	1662	**0.5**
	E5 Large	0.033	0.133	0.3	0.111	0.037	0.022	2342	2050	0.111
	UAE Large	**0.133**	0.333	0.667	0.1	0.056	0.039	968	762	0.25
	Snowflake Arctic L	0.033	0.167	0.433	0.091	0.029	0.018	1818	1296	0.091
	QWEN2	0	0.2	0.267	0	0.011	0.011	2164	1651	0.007
	Stella	0.1	0.267	0.433	0.033	0.031	0.025	2393	1124	0.027
	Mistral	0.1	0.2	0.467	0.084	0.051	0.031	1328	1059	0.125
Venture capital	MiniLM	0.08	0.267	0.507	**0.594**	**0.245**	**0.165**	1322	996	**1**
	BGE Large	0.1	0.28	0.447	0.212	0.151	0.122	1611	1152	**1**
	BGE M3	0.06	0.22	0.387	0.478	0.189	0.134	2531	1558	**1**
	E5 Large	**0.107**	0.247	0.367	0.245	0.161	0.129	2283	1626	0.167
	UAE Large	**0.107**	0.293	0.5	0.28	0.176	0.138	1416	993	**1**
	Snowflake Arctic L	0.06	0.18	0.347	0.152	0.095	0.075	2137	1431	0.1
	QWEN2	0.007	0.067	0.153	0.031	0.019	0.021	3695	2785	0.031
	Stella	0.033	0.147	0.227	0.251	0.101	0.08	3812	2719	**1**
	Mistral	0.093	**0.353**	**0.573**	0.12	0.116	0.109	**1608**	**794**	0.056
Relational learning	MiniLM	**0.652**	**0.913**	0.957	**0.345**	**0.277**	**0.266**	**157**	**45**	**1**
	BGE Large	0.522	**0.913**	0.957	0.273	0.193	0.186	216	100	**1**
	BGE M3	0.261	0.609	0.783	0.11	0.071	0.061	794	311	0.25
	E5 Large	0.348	0.696	0.87	0.285	0.164	0.137	444	267	**1**
	UAE Large	0.522	**0.913**	0.957	0.335	0.233	0.224	198	80	**1**
	Snowflake Arctic L	0.304	0.87	**1**	0.295	0.144	0.129	244	186	**1**
	QWEN2	0	0.087	0.348	0	0.008	0.008	2926	2469	0.009
	Stella	0.261	0.652	0.783	0.229	0.123	0.107	1393	246	**1**
	Mistral	0.348	0.783	0.87	0.14	0.093	0.087	399	184	0.25
Ambient assisted living	MiniLM	0.037	**0.102**	**0.204**	0.072	0.047	0.035	4373	3168	0.091
	BGE Large	0.019	0.074	0.157	0.101	0.04	0.029	5446	4397	0.111
	BGE M3	0.009	0.046	0.13	0.019	0.018	0.015	6034	5488	0.019
	E5 Large	0	0.028	0.074	0	0.007	0.008	5943	4521	0.006
	UAE Large	0.019	0.083	0.148	0.038	0.02	0.02	5619	4613	0.045
	Snowflake Arctic L	0.009	0.065	0.111	0.011	0.013	0.013	6725	5542	0.011
	QWEN2	0.009	0.037	0.056	0.012	0.01	0.01	8254	7112	0.012
	Stella	0.028	0.056	0.139	0.036	0.03	0.021	6573	4210	0.033
	Mistral	**0.046**	**0.102**	**0.204**	**0.12**	**0.07**	**0.047**	**3921**	**2827**	**0.25**
10-m walks	MiniLM	0.5	0.818	0.955	0.2	0.151	0.135	218	122	0.2
	BGE Large	0.409	0.864	0.955	0.186	0.123	0.114	251	140	**0.5**
	BGE M3	0.455	0.864	0.909	**0.407**	**0.247**	**0.235**	285	129	**0.5**
	E5 Large	0.364	0.818	**1**	0.309	0.179	0.152	240	133	0.25
	UAE Large	0.409	0.864	0.955	0.186	0.122	0.113	262	173	**0.5**
	Snowflake Arctic L	0.5	0.818	0.909	0.322	0.224	0.205	274	106	0.25
	QWEN2	0.136	0.636	0.727	0.097	0.049	0.046	874	276	0.2
	Stella	0.318	0.727	0.864	0.074	0.065	0.059	473	186	0.048
	Mistral	**0.682**	**0.955**	**1**	0.224	0.192	0.185	**103**	**68**	0.333

*R@K* Recall@K, *AP@K* Average Precision@K, *AR* average rank, *MR* median rank, *RR* reciprocal rank

Bold numbers = best choices within the table

In Table 7, we summarize the results obtained using the few-shot prompting technique. The results for the Driver Healthcare Monitor dataset are consistent with those from the zero-shot approach, with BGE Large and BGE M3 emerging as the top performers. In the Venture Capital dataset, Mistral achieves the highest recall values, while MiniLM excels in precision at smaller cutoffs, and Snowflake Arctic L shows strong precision at larger cutoffs.

UAE Large performs exceptionally well for the Relational Learning dataset, achieving near-perfect recall and the highest scores across rank and precision metrics. This dataset demonstrates strong performance across multiple models, similar to the zero-shot approach. Finally, Mistral stands out across most metrics in the last two datasets, outperforming other models by a substantial margin. The only exception is BGE M3, which excels in average precision.

**Table 7:** Few shot results

Dataset	Model	R@100	R@500	R@1000	AP@100	AP@500	AP@1000	AR	MR	RR
Driver healthcare monitor	MiniLM	0.067	0.133	0.233	0.11	0.06	0.037	2387	1901	0.167
	BGE Large	**0.1**	**0.433**	**0.7**	0.193	0.069	0.052	**921**	**592**	0.5
	BGE M3	0.067	0.233	0.333	**0.562**	**0.172**	**0.124**	1984	1665	**1**
	E5 Large	0.067	0.167	0.267	0.127	0.063	0.043	2481	2524	0.143
	UAE Large	**0.1**	0.367	0.667	0.385	0.124	0.078	1000	712	**1**
	Snowflake Arctic L	0.033	0.133	0.3	0.2	0.059	0.031	2163	1414	0.2
	QWEN2	0	0.067	0.067	0	0.009	0.009	8430	9613	0.006
	Stella	0	0.267	0.333	0	0.017	0.016	2995	2262	0.008
	Mistral	0.067	0.3	0.533	0.061	0.028	0.023	1096	809	0.091
Venture capital	MiniLM	0.073	0.413	0.607	0.318	0.155	0.139	**1192**	736	**1**
	BGE Large	0.073	0.273	0.507	0.224	0.134	0.109	1634	992	0.333
	BGE M3	0.047	0.233	0.48	**0.486**	0.169	0.121	2021	1102	**1**
	E5 Large	0.08	0.227	0.413	0.442	0.209	0.144	1921	1217	**1**
	UAE Large	0.087	0.32	0.567	0.394	0.188	0.145	1388	883	**1**
	Snowflake Arctic L	0.093	0.233	0.44	0.43	**0.225**	**0.152**	1856	1212	**1**
	QWEN2	0.013	0.047	0.14	0.038	0.024	0.023	4675	3546	0.038
	Stella	0.08	0.28	0.413	0.116	0.106	0.095	2796	1586	0.067
	Mistral	**0.107**	**0.42**	**0.66**	0.147	0.148	0.136	1388	**630**	0.167
Relational learning	MiniLM	0.609	0.913	0.957	0.228	0.18	0.173	194	82	**1**
	BGE Large	0.652	0.913	0.957	0.32	0.26	0.25	168	78	**1**
	BGE M3	0.304	0.609	0.783	0.216	0.132	0.108	738	358	**1**
	E5 Large	0.348	0.696	0.87	0.301	0.179	0.149	406	214	**1**
	UAE Large	**0.696**	**0.957**	0.957	**0.376**	**0.305**	**0.305**	**135**	**67**	**1**
	Snowflake Arctic L	0.565	0.87	**1**	0.258	0.202	0.181	156	88	**1**
	QWEN2	0	0.087	0.174	0	0.003	0.004	3693	2801	0.002
	Stella	0.435	0.739	0.826	0.206	0.151	0.138	1174	137	**1**
	Mistral	0.435	0.696	0.87	0.197	0.145	0.123	361	149	0.25
Ambient assisted living	MiniLM	0.009	0.093	0.148	0.037	0.022	0.02	4819	3582	0.037
	BGE Large	0.028	0.102	0.194	0.068	0.036	0.03	4749	3666	0.111
	BGE M3	0.009	0.065	0.111	0.016	0.015	0.015	5615	4858	0.016
	E5 Large	0	0.028	0.083	0	0.009	0.009	5915	4316	0.008
	UAE Large	0.028	0.093	0.167	0.04	0.027	0.023	4966	3775	0.053
	Snowflake Arctic L	0.009	0.065	0.13	0.013	0.021	0.017	5312	3944	0.013
	QWEN2	0.009	0.037	0.056	0.013	0.013	0.011	8613	7188	0.013
	Stella	0.037	0.083	0.185	0.075	0.049	0.033	5782	3926	0.077
	Mistral	**0.046**	**0.139**	**0.231**	**0.148**	**0.07**	**0.053**	**3438**	**2493**	**0.2**
10-m walks	MiniLM	0.409	0.818	0.955	0.125	0.1	0.091	234	147	0.2
	BGE Large	0.455	0.864	0.955	0.156	0.108	0.101	264	185	**0.5**
	BGE M3	0.5	0.818	0.864	**0.39**	**0.263**	**0.251**	313	115	0.5
	E5 Large	0.409	0.818	**1**	0.155	0.109	0.095	243	146	0.25
	UAE Large	0.409	0.864	0.955	0.166	0.11	0.103	263	147	**0.5**
	Snowflake Arctic L	0.545	0.864	0.909	0.349	0.248	0.237	259	87	**0.5**
	QWEN2	0.091	0.227	0.455	0.182	0.085	0.048	1375	1181	0.333
	Stella	0.182	0.636	0.773	0.065	0.053	0.047	692	215	0.071
	Mistral	**0.636**	**0.955**	**1**	0.26	0.206	0.199	**119**	**61**	**0.5**

*R@K* Recall@K, *AP@K* Average Precision@K, *AR* average rank, *MR* median rank, *RR* reciprocal rank

Bold numbers = best choices within the table

Finally, we conclude our results with the performance of the models using the prompt chaining technique, as outlined in Table 8. Compared to the zero-shot and few-shot techniques, we observe greater variability in model performance, particularly in the first two datasets. Despite this variability, BGE Large, BGE M3, and UAE Large stand out as the top performers for these datasets.

**Table 8:** Prompt chaining results

Dataset	Model	R@100	R@500	R@1000	AP@100	AP@500	AP@1000	AR	MR	RR
Driver healthcare monitor	MiniLM	0.033	0.1	0.133	0.011	0.011	0.009	2721	2094	0.011
	BGE Large	0.1	**0.4**	**0.6**	0.177	0.063	0.049	**1068**	**768**	0.333
	BGE M3	0.1	0.3	0.367	0.15	0.062	0.053	1720	1425	0.25
	E5 Large	0	0.1	0.2	0	0.006	0.006	3108	3146	0.008
	UAE Large	**0.133**	0.267	0.533	**0.203**	**0.113**	**0.065**	1203	960	**0.5**
	Snowflake Arctic L	0.033	0.1	0.233	0.067	0.03	0.017	2195	1781	0.067
	QWEN2	0.033	0.1	0.133	0.013	0.01	0.009	3602	3154	0.013
	Stella	0.033	0.3	0.433	0.022	0.023	0.021	2300	1389	0.022
	Mistral	0.067	0.3	0.533	0.153	0.055	0.039	1109	785	0.125
Venture capital	MiniLM	0.087	**0.4**	**0.66**	0.118	0.124	0.12	**1045**	**669**	0.091
	BGE Large	0.06	0.227	0.46	0.234	0.123	0.097	1613	1214	**1**
	BGE M3	0.08	0.273	0.473	**0.331**	**0.169**	**0.13**	1962	1083	**1**
	E5 Large	0.08	0.227	0.407	0.285	0.158	0.117	1903	1262	**1**
	UAE Large	**0.093**	0.253	0.56	0.259	0.153	0.117	1391	846	**1**
	Snowflake Arctic L	0.073	0.193	0.32	0.121	0.096	0.079	2078	1435	0.25
	QWEN2	0	0.02	0.067	0	0.006	0.008	6514	6190	0.003
	Stella	0.053	0.28	0.453	0.08	0.092	0.086	2461	1275	0.053
	Mistral	0.047	0.267	0.447	0.056	0.078	0.075	1922	1133	0.02
Relational learning	MiniLM	0.435	0.783	**0.957**	0.25	0.176	0.15	259	128	**1**
	BGE Large	**0.522**	**0.913**	0.913	0.262	0.187	0.187	214	92	**1**
	BGE M3	0.174	0.522	0.826	0.071	0.046	0.037	832	465	0.091
	E5 Large	0.304	0.696	0.913	0.168	0.099	0.082	424	335	0.2
	UAE Large	**0.522**	**0.913**	**0.957**	**0.34**	**0.236**	**0.227**	**186**	**85**	**1**
	Snowflake Arctic L	0.391	**0.913**	**0.957**	0.29	0.161	0.155	217	179	**1**
	QWEN2	0	0.043	0.087	0	0.003	0.002	6059	6285	0.003
	Stella	0.174	0.565	0.739	0.109	0.055	0.047	1468	400	0.25
	Mistral	0.348	0.652	0.826	0.107	0.078	0.068	463	258	0.077
Ambient assisted living	MiniLM	**0.056**	**0.13**	0.194	0.063	0.048	**0.041**	**4230**	3202	0.062
	BGE Large	**0.056**	0.12	**0.259**	0.062	**0.049**	0.037	4363	3448	0.038
	BGE M3	0.009	0.074	0.157	0.01	0.013	0.015	5531	5024	0.01
	E5 Large	0.046	0.12	0.213	0.048	0.035	0.03	4524	**2689**	0.053
	UAE Large	0.019	0.111	0.241	0.025	0.023	0.025	4622	3492	0.019
	Snowflake Arctic L	0.019	0.074	0.12	0.017	0.018	0.016	5831	4209	0.011
	QWEN2	0	0.009	0.019	0	0.002	0.003	9808	8927	0.002
	Stella	0.019	0.037	0.139	0.02	0.015	0.013	7119	5244	0.016
	Mistral	0.037	0.083	0.176	**0.075**	**0.049**	0.033	4618	3536	**0.125**
10-m walks	MiniLM	0.5	0.955	**1**	0.149	0.115	0.111	175	107	0.125
	BGE Large	0.364	0.864	**1**	0.184	0.121	0.109	220	139	0.5
	BGE M3	0.455	0.864	0.955	**0.353**	**0.221**	**0.202**	239	120	**1**
	E5 Large	0.409	0.864	**1**	0.22	0.142	0.126	220	120	0.25
	UAE Large	0.364	0.864	0.955	0.193	0.124	0.115	227	145	0.5
	Snowflake Arctic L	0.455	0.818	0.909	0.317	0.209	0.192	238	139	0.25
	QWEN2	0.136	0.545	0.727	0.351	0.113	0.09	786	406	**1**
	Stella	0.318	0.636	0.909	0.104	0.083	0.066	442	181	0.25
	Mistral	**0.545**	**1**	**1**	0.29	0.202	0.202	**110**	**71**	0.5

*R@K* Recall@K, *AP@K* Average Precision@K, *AR* average rank, *MR* median rank, *RR* reciprocal rank

Bold numbers = best choices within the table

For the Relational Learning dataset, UAE Large remains the best performer, maintaining consistency with the results from the previous techniques. In contrast, multiple models achieve top scores across different metrics for the Ambient Assisted Living dataset, but even the best-performing models show only modest results. The final dataset, 10-m walks, has results consistent with previous techniques, with Mistral and BGE M3 continuing to lead across most metrics.

Overall, we observe notable differences in performance across datasets, regardless of the prompt engineering technique or embedding model used. Interestingly, while the top-performing models vary across different datasets, the same models tend to lead within each dataset across various prompt techniques consistently. This suggests that certain embedding models are better aligned with the semantic and domain characteristics of specific datasets. The Relational Learning and 10-m walks datasets consistently demonstrate high performance across metrics. The selection process in these datasets includes more general articles that broadly reflect the set of semantic properties used in the query vector. In contrast, the Ambient Assisted Living dataset shows lower performance levels, likely due to its narrow focus on highly specific articles that correspond to only a small subset of the defined semantic properties. The remaining two datasets fall somewhere in between, with performance varying depending on the technique and model used.

## Discussion

In this section, we will reflect on the key findings of our research, analyzing trends observed in the performance of the different prompt engineering techniques, embedding models, and vector indexes. By examining the results in detail, we aim to answer the three main questions posed in this study. Specifically, we will identify the embedding models that demonstrate the most consistent and reliable performance, explore the effectiveness of GenAI in query reformulation, and highlight the trade-offs between computational efficiency and retrieval performance when comparing vector indexes.

### Massive text embeddings for document retrieval

The first focus of our investigation was to identify the best-performing massive embedding model for document retrieval, given our selection of models that vary in size and training data. Initial observations reveal a variation in top performers depending on the dataset and the prompt engineering technique applied. However, four models (BGE Large, UAE Large, MiniLM, and Mistral) consistently rank among the top performers in various scenarios. To address our research question of selecting an embedding model that performs robustly across different datasets and domains, we proceed with a more generalized evaluation using ranking-based assessment.

Our evaluation of the best-performing, domain-independent embedding model follows a multi-step ranking framework. We use dense ranking throughout, where models with identical scores share the same rank, and the next model receives the subsequent position without skipping ranks. First, we individually rank the models for each metric within each dataset. Next, to obtain a dataset-independent ranking, we aggregate the ranks for each metric across all datasets and apply another dense ranking. This provides a ranked list of embedding models per prompt engineering technique and metric, as presented in Table 9. Finally, we aggregate the ranking positions across all metrics using a simple average to establish an overall ranking, assigning equal weight to each metric. We then apply the dense ranking again, producing a final ranking for each prompt engineering technique.

**Table 9:** Rankings of embedding models

Technique	Model	R@100	R@500	R@1000	AP@100	AP@500	AP@1000	AR	MR	RR	Final rank
Ground truth	UAE Large	2	1	1	4	1	1	1	2	3	1
	BGE Large	1	2	2	2	1	2	2	1	3	1
	MiniLM	3	3	3	6	3	5	3	3	2	2
	Mistral	4	4	4	5	4	3	3	4	2	3
	BGE M3	5	5	6	1	2	4	4	6	1	4
	Snowflake Arctic L	3	6	5	6	5	6	5	5	5	5
	Stella	6	8	7	3	2	4	6	8	4	6
	E5 Large	6	7	6	7	6	7	5	7	6	7
	QWEN2	7	9	8	8	7	8	7	9	7	8
Zero shot	MiniLM	2	4	4	1	1	1	1	2	5	1
	UAE Large	2	1	3	3	3	2	3	4	2	2
	BGE Large	3	2	2	4	5	4	3	3	1	3
	Mistral	1	3	1	7	4	5	2	1	4	4
	BGE M3	6	6	6	2	2	3	6	7	3	5
	Snowflake Arctic L	5	5	5	6	7	7	4	5	7	6
	E5 Large	4	8	7	5	6	6	5	7	6	7
	Stella	7	7	8	8	8	8	7	6	6	8
	QWEN2	8	9	9	9	9	9	8	8	8	9
Few shot	UAE Large	2	1	3	2	1	1	2	2	1	1
	Mistral	1	2	1	6	4	4	1	1	3	2
	BGE Large	3	3	2	4	3	3	3	3	1	3
	Snowflake Arctic L	3	5	5	3	2	2	5	5	3	4
	MiniLM	4	3	4	7	6	5	4	4	4	5
	BGE M3	6	6	7	1	3	4	7	6	2	6
	E5 Large	6	7	7	5	5	6	6	8	5	7
	Stella	5	4	6	8	7	7	8	7	6	8
	QWEN2	7	8	8	9	8	8	9	9	7	9
Prompt chaining	UAE Large	2	3	1	1	1	1	2	1	1	1
	BGE Large	3	2	1	2	2	2	1	1	1	2
	MiniLM	1	1	2	6	5	3	2	1	3	3
	Mistral	4	3	3	4	4	4	3	2	2	4
	BGE M3	5	5	4	4	3	3	6	4	2	5
	E5 Large	5	4	5	5	5	5	4	3	4	6
	Snowflake Arctic L	4	7	7	3	4	5	5	5	3	7
	Stella	6	6	6	7	6	6	7	6	5	8
	QWEN2	7	8	8	8	7	7	8	7	6	9

*R@K* Recall@K, *AP@K* Average Precision@K, *AR* average rank, *MR* median rank, *RR* reciprocal rank

The results of our evaluation framework indicate that the UAE Large embedding model consistently emerges as the top performer across all prompt engineering techniques, except in the zero-shot setting, where it ranks second behind MiniLM. BGE Large also demonstrates strong, consistent performance, consistently placing within the top three for all techniques and even sharing the top spot with UAE Large in the ground truth rankings. MiniLM and Mistral follow as solid contenders, while Stella and QWEN2 consistently occupy the two lowest positions, regardless of the technique. We observe that the highest-performing models employ fine-tuning strategies specifically targeting tasks such as information retrieval, document ranking, or semantic similarity. On the other hand, the lower-ranked models, despite having significantly more parameters, employ fine-tuning strategies for broader or unrelated tasks, which explains their underperformance.

In a similar manner, Table 10 presents the average rankings of the embedding models, calculated according to the approach proposed by [67]. We prioritize the Recall@500 metric for this analysis because high recall is essential in literature reviews where the objective is to retrieve as many relevant documents as possible for assessment. The cutoff at 500 reflects a practical limit, as reviewing the top 500 documents is typically a manageable and effective scope in academic settings. The results reinforce the findings from the previous analysis, with UAE Large and BGE Large taking the top 2 positions.

**Table 10:** Average rankings of embedding models (Friedman) for Recall@500

Embedding model	Ranking
UAE Large	**2.333**
BGE Large	2.567
Mistral	3.400
MiniLM	3.933
Snowflake Arctic L	5.600
BGE M3	5.633
Stella	6.367
E5 Large	6.733
QWEN2	8.433

Bold numbers = best choices within the table

When considering additional parameters such as time and memory efficiency, we observe that UAE Large and BGE Large require 5 to 6 times more time than MiniLM for the initial embedding of documents. Additionally, with vector dimensions approximately 2.7 times larger than those of MiniLM, the indexes created from UAE Large and BGE Large embeddings are also 2.7 times larger, potentially impacting query times. There is no difference between UAE Large and BGE Large, as both models exhibit comparable embedding generation times and index sizes.

Our findings indicate that information-retrieval-specific embedding models generally outperform general-purpose models for automated document retrieval in literature reviews. Among the evaluated models, UAE Large stands out as the best overall choice for this purpose. However, MiniLM offers a practical alternative due to its efficiency in scenarios where time or memory constraints are critical.

### Human versus GenAI in query reformulation for document retrieval

The second goal of our study was to compare the effectiveness of human experts and generative AI in transforming a researcher’s initial idea into a well-defined query for automated document retrieval in literature reviews. Our results show that GenAI-generated queries consistently outperformed those formulated by human experts across all evaluation metrics for datasets such as Driver Healthcare Monitoring and 10-m walks. Furthermore, the differences were minimal for the Venture Capital and Relational Learning datasets, with GenAI showing slight improvements or performing comparably to the human queries. However, for the Ambient Assisted Living dataset, the AI-generated queries underperformed those of the human-generated ones. We attribute this underperformance to the significantly more refined query construction, where the expert researchers iterated over the process of defining semantic properties more than 10 times to align them closely with the requirements. The other datasets involved only one or a few refinement iterations. We proceed with a more in-depth statistical and numerical analysis to gain a deeper understanding of these variations.

We conducted a dataset-specific pairwise comparison between the techniques, including the ground truth, to gain further insights. For each pair of techniques, we took the result obtained by a specific embedding model on a given metric for *Technique A* and compared it to the result for the same model and metric for *Technique B*. If Technique A outperformed Technique B, we incremented the count for Technique A, and vice versa. These comparative results are presented in Table 11.

**Table 11:** Prompt engineering technique pairwise comparison

Dataset	GT	ZS	GT	FS	GT	PC	ZS	FS	ZS	PC	FS	PC
Driver healthcare monitor	10	**66**	13	**64**	19	**54**	**38**	34	**49**	25	**47**	28
Venture capital	30	**46**	12	**60**	**45**	34	22	**56**	**46**	32	**56**	18
Relational learning	21	**53**	16	**60**	32	**46**	16	**42**	**51**	16	**64**	6
Ambient assisted living	**68**	9	**65**	14	**73**	5	18	**50**	37	**39**	**43**	36
10-m walks	0	**81**	0	**81**	0	**81**	**39**	25	26	**38**	18	**51**
**Total**	129	**255**	106	**279**	169	**220**	133	**207**	**209**	150	**228**	139

*GT* ground truth, *ZS* zero-shot, *FS* few-shot, *PC* prompt chaining

Bold numbers = best choices within the table

Our findings confirm that, for four out of five datasets, both zero-shot and few-shot techniques outperform the ground truth, except for the Ambient Assisted Living dataset. When directly comparing zero-shot and few-shot techniques, we observe no statistically significant difference, although few-show generally shows a slight advantage. However, the key observation is that both techniques consistently outperform the ground truth.

**Fig. 6:**
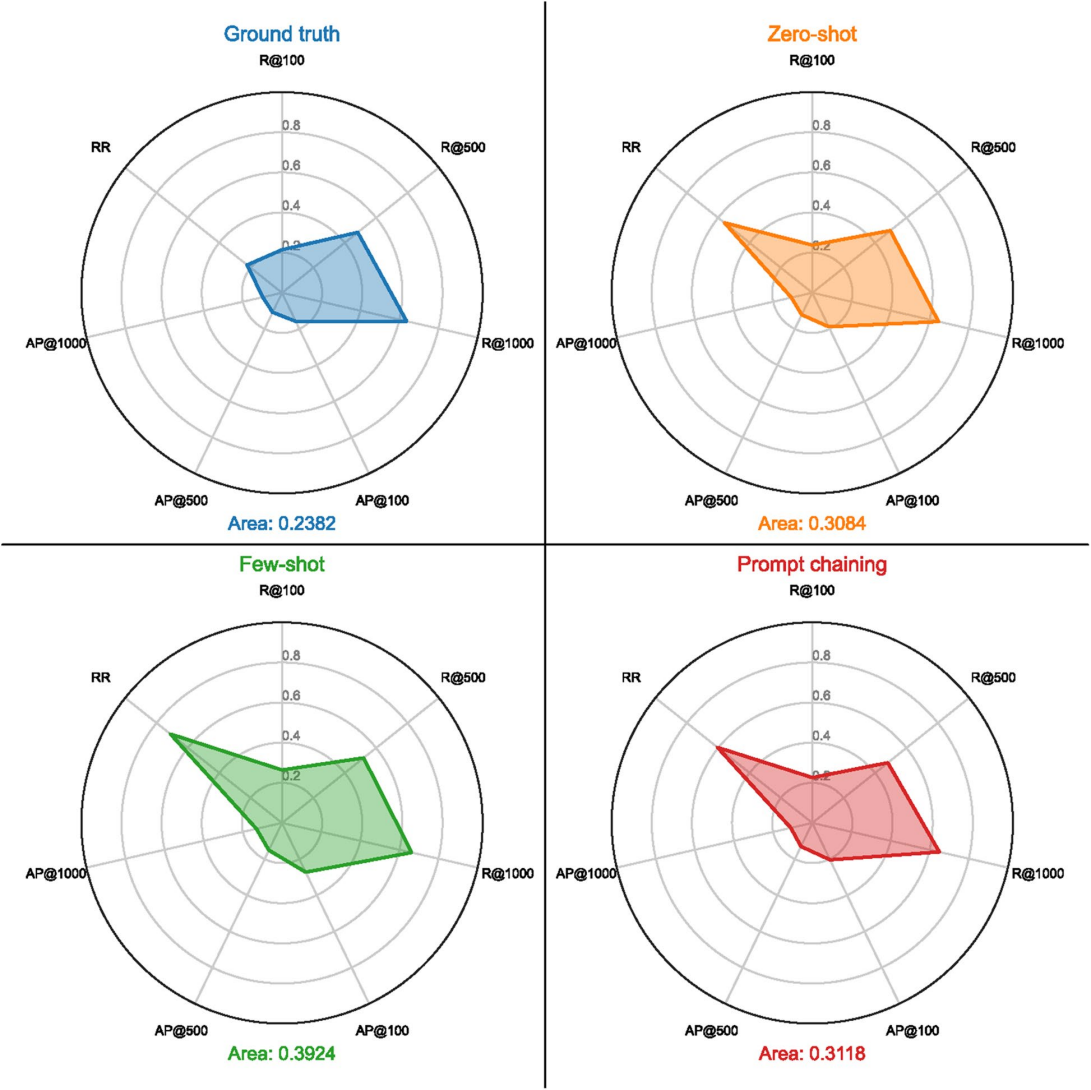
Prompt engineering technique radar chart

Since we concluded that UAE Large is the embedding model that is the top performer, we selected its result to compare the ground truth with the three prompt engineering techniques. Then, we averaged the results for each metric across all datasets, resulting in a single value per metric for each prompt engineering technique, allowing for a more general comparison that is not dataset-specific. Using the metrics that provide results between 0 and 1, we constructed radar charts to visualize the performance of each technique, with each metric represented as a variable, as shown in Fig. 6.

From the figure, it is evident that the polygon generated for the few-shot prompt engineering techniques is larger than the others, indicating better overall performance using the UAE Large model. This observation is further supported by the area each polygon covers: 0.39 for few-shot, 0.31 for prompt chaining, 0.30 for zero-shot, and 0.23 for the ground truth.

In summary, regarding the choice between human expertise and GenAI for query reformulation, our findings suggest that GenAI is generally the preferable option due to its superior performance and the ability to reduce manual labor and time investment. However, in cases where human experts are willing and able to invest substantial time in multiple iterations and careful tuning of semantic criteria, they may outperform GenAI, as observed in the Ambient Assisted Living dataset. Therefore, as a recommended approach, GenAI could be used for the initial query formulation, with human experts potentially involved in a subsequent evaluation phase, thus minimizing their effort and time commitment.

### Vector indexes trade-offs

In the final part of our research, we examine the trade-offs between speed, memory efficiency, and retrieval accuracy in different vector indexing techniques. Although the flat index serves as our baseline and performs well within the scope of our current datasets, scalability considerations become essential as the datasets grow. We recognize the potential need for faster response times for larger datasets, initial domain-specific collections of documents, or a high volume of queries to enhance retrieval robustness. In such scenarios, exploring alternative indexing strategies could offer substantial gains.

When comparing the IVF index to the Flat index, we begin by examining memory efficiency. Both indexes are similar in size across all datasets, with the IVF index requiring only minimal additional memory to store centroid metadata used in the query phase. For the time parameter, two aspects are considered: initial build and training time, and response time. Both indexes complete the initial vector build and training in less than a second, making this factor negligible. However, in terms of query response time, the IVF index performs significantly faster, returning the top 100 closest documents around 18 times faster than the Flat index, and the top 1000 documents about 9 times faster. While these differences are small in single-query scenarios because we measure in milliseconds, they become impactful when handling large query volumes. For instance, executing 1000 queries would take approximately 18 s with the Flat index, compared to just 1–2 s with the IVF index. Lastly, regarding retrieval accuracy, the IVF index performs comparably to the Flat index, with only a slight decrease overall, and in some cases, the IVF index even shows better results.

The primary advantage of the PQ index lies in memory compression, consuming roughly 20 times less memory than the Flat index across all datasets. However, this memory efficiency comes at the expense of build and training time, where PQ takes approximately 20 times longer than the Flat index, with figures around 10 s for 12,000 documents and up to 20 s for 25,000 documents. Despite the initial requirements, PQ compensates with faster query response times, performing around 9 times faster in retrieval speed. This advantage becomes meaningful in high-query scenarios where, with 1000 or more queries, PQ’s reduced response time offsets its slower initial build. As for retrieval accuracy, PQ performs similarly to the IVF index, maintaining results comparable to the Flat index but with a slight reduction in overall performance. Comprehensive results for both IVF and PQ indexes are provided in the supplementary materials.

Our findings suggest that the Flat index well supports the current requirements, making it a suitable and sufficient choice for this use case. However, we recognize that if future requirements demand greater scalability, whether due to an increase in the number of documents or queries, the IVF and PQ indexes provide viable alternatives that offer faster response times and comparable retrieval performance.

### Limitations

In this study, embeddings and similarity search were computed using only paper titles and abstracts rather than full-text articles. This is a limitation because studies with short or less informative abstracts may be ranked lower even when the full text is relevant, while studies whose abstracts mention many key terms may be ranked higher even if the full text is not closely aligned. As a result, the reported performance may not fully generalize to full-text screening or later stages of evidence synthesis such as data extraction. We made this design choice because titles and abstracts are broadly accessible, whereas full texts are often unavailable due to paywalls or other access restrictions, reflecting a common constraint in human review processes.

LLM based query reformulation also introduces reproducibility limitations. Even when using the same prompts and the same model, generated outputs can vary across runs due to stochastic decoding and implementation details. Consequently, reproducing the query reformulation step may not produce exactly the same performance numbers. To improve transparency and support replication and reproduction, we report the full prompts used in the study and provide the complete LLM conversation logs for the query reformulation step in our code and data repository.

## Conclusion

In this study, we investigated the potential of generative AI in query reformulation, evaluated the effectiveness of massive text embedding models in enhancing automated document retrieval for literature reviews, and examined the trade-offs between speed, memory, and retrieval accuracy offered by different vector indexes. All experiments were conducted on real-world datasets curated through our NLP toolkit that supports the paper selection phase in literature reviews.

Our findings indicate that GenAI-generated queries, specifically zero-shot and few-shot prompting, generally outperform the queries designed by humans. This suggests that GenAI can reduce the manual labor involved in query formulation, providing researchers with efficient, high-quality assistance in formulating suitable queries.

We also observed that embedding models designed or fine-tuned specifically for information retrieval consistently outperformed general-purpose models. UAE Large emerged as the most reliable performer across diverse datasets spanning healthcare, finance, social care, and machine learning domains.

Finally, our evaluation of vector indexes shows that the flat index is a sufficient choice for storing and querying documents in the current context of automated literature reviews. However, the IVF and PQ indexes present viable alternatives for scalability if the dataset size or query volume increases significantly.

## Data Availability

The data and the code we utilize in this study are openly available on https://gitlab.com/mitrovg/article-analysis-study/-/tree/main.
